# Supplementary material for: Asynchronous magnetic resonance elastography: Shear wave speed reconstruction using noise correlation of incoherent waves
Source: Magn Reson Med. 2022 Oct 27;89(3):990–1001. doi: 10.1002/mrm.29502 (PMC9792433; doi:10.1002/mrm.29502)
Supplement: Supplementary file 1 — DATA S1 MATLAB code used to generate the numerical simulations of Figure 1 [file MRM-89-990-s002.zip › k-Wave/helpfiles/example_ivp_homogeneous_medium.html]

Homogeneous Propagation Medium Example (k-Wave)


# Homogeneous Propagation Medium Example

This example provides a simple demonstration of using k-Wave for the simulation and detection of the pressure field generated by an initial pressure distribution within a two-dimensional homogeneous propagation medium.

- Open the file in the MATLAB Editor
- Run the file in MATLAB

## Contents

- Creating the k-space grid
- Avoiding the perfectly matched layer
- Defining the medium properties
- Defining the initial pressure distribution
- Defining the sensor mask
- Running the simulation

## Creating the k-space grid

The simulation functions in k-Wave require four input structures. These define the properties of the computational grid, the material properties of the medium, the properties and locations of any acoustic sources, and the properties and locations of the sensor points used to record the evolution of the pressure and velocity fields over time. Starting with the computational grid, the simulations are performed on a regular Cartesian mesh (for users with a background in finite-element methods, this is analogous to a structured mesh containing identical rectangular elements). The medium discretisation is performed using `kWaveGrid`. Both the total number of grid points (Nx, Ny) as well as the spacing between the grid points (dx, dy) in each Cartesian direction are used to compute the discretisation, and an object of the `kWaveGrid` class is returned. As the numerical techniques used in k-Wave are based heavily on the fast Fourier transform (FFT), the simulations will be fastest when the number of grid points in each direction is given by a power of two or has small prime factors (see `fft2` and `checkFactors`).

```
% create the computational grid
Nx = 128;           % number of grid points in the x (row) direction
Ny = 128;           % number of grid points in the y (column) direction
dx = 0.1e-3;        % grid point spacing in the x direction [m]
dy = 0.1e-3;        % grid point spacing in the y direction [m]
kgrid = kWaveGrid(Nx, dx, Ny, dy);
```

The object `kgrid` contains numerous properties, including matrices of the computational wavenumbers (`kgrid.kx`, `kgrid.ky`, and `kgrid.k`) and Cartesian coordinates of the grid points (`kgrid.x`, `kgrid.y`). These properties are used by many k-Wave functions.

## Avoiding the perfectly matched layer

When the acoustic waves reach the edge of the computational domain, they are absorbed by a special type of anisotropic absorbing boundary layer known as a perfectly matched layer (PML). The effects of the layer can be seen by watching what happens to the propagating waves as they get close to the edge of the computational domain. By default, this layer occupies a strip of 20 grid points (10 grid points in 3D) around the edge of the domain *inside* the computational domain specified using `kWaveGrid`. To avoid strange effects, care must be taken not to place the source or sensor points inside this layer. Alternatively, the perfectly matched layer can be set to be *outside* the computational domain set by the user. See Controlling The Absorbing Boundary Layer Example for more detailed instructions on how to modify the properties and position of the perfectly matched layer.

## Defining the medium properties

For a homogeneous medium, the sound speed is set as a scalar value in SI units (i.e., metres per second). Power law acoustic absorption can also be optionally set by assigning values to `medium.alpha_coeff` and `medium.alpha_power`. These respectively correspond to the power law coefficient or prefactor in units of dB/(MHz^y cm) and the power law exponent or power y. It is useful to note that for a homogeneous medium, the computation of the acoustic pressure is not dependent on the ambient density. Consequently, if no velocity inputs or outputs are required, `medium.density` does not need to be specified. For modelling nonlinear effects, `medium.BonA` should also be specified. In this case, k-Wave includes a number of additional nonlinear terms in the discrete governing equations.

```
% define the properties of the propagation medium
medium.sound_speed = 1500;  % [m/s]
medium.alpha_coeff = 0.75;  % [dB/(MHz^y cm)]
medium.alpha_power = 1.5;
```

The grid spacing (`dx` and `dy`) and the sound speed govern the maximum frequency that the simulation grid is able to propagate. This frequency is printed to the command line when the simulation runs, and can be calculated (for each Cartesian direction) by `f_max_x = medium.sound_speed/(2*dx)`.

## Defining the initial pressure distribution

The initial pressure distribution is set as a matrix which contains the initial pressure values for each grid point. This matrix must be the same size as the medium discretisation defined by the computational grid (i.e., it must have `Nx` rows and `Ny` columns). Several functions are included in the toolbox for the creation of simple geometric shapes. In this example, the function `makeDisc` is used to create an initial pressure distribution in the shape of two small discs with different diameters. This distribution is assigned to the `p0` field of the `source` structure. There are no restrictions on `source.p0` except that it must be the same size as the computational grid.

```
% create initial pressure distribution using makeDisc
disc_magnitude = 5; % [Pa]
disc_x_pos = 50;    % [grid points]
disc_y_pos = 50;    % [grid points]
disc_radius = 8;    % [grid points]
disc_1 = disc_magnitude * makeDisc(Nx, Ny, disc_x_pos, disc_y_pos, disc_radius);

disc_magnitude = 3; % [Pa]
disc_x_pos = 80;    % [grid points]
disc_y_pos = 60;    % [grid points]
disc_radius = 5;    % [grid points]
disc_2 = disc_magnitude * makeDisc(Nx, Ny, disc_x_pos, disc_y_pos, disc_radius);

source.p0 = disc_1 + disc_2;
```

## Defining the sensor mask

The sensor mask defines the positions within the computational domain where the pressure field is recorded at each time-step. The sensor mask can be given in one of three ways:

1. A binary matrix which specifies the grid points that record the data (see the Using A Binary Sensor Mask Example).
2. The grid coordinates of two opposing corners of a line (1D), rectangle (2D) or cuboid (3D) (see the Defining A Sensor Mask By Opposing Corners Example).
3. A set of Cartesian coordinates lying within the dimensions of the computational domain.

In 2D, Cartesian coordinates must be specified as a 2 x N matrix, where the Cartesian origin is assumed to be in the center of the grid. Here the function `makeCartCircle` is used to set the sensor mask to the Cartesian coordinates of a set of evenly spaced points on a circle.

```
% define a centered circular sensor
sensor_radius = 4e-3;   % [m]
num_sensor_points = 50;
sensor.mask = makeCartCircle(sensor_radius, num_sensor_points);
```

A plot of the initial pressure distribution and the sensor mask is given below. By default, the pressure at the Cartesian points in 2D is computed at each time step using linear interpolation.


## Running the simulation

The computation is started by calling the function `kspaceFirstOrder2D` with the four input structures defined above. By default, a visualisation of the propagating wave-field and a status bar are displayed, with frame updates every ten time-steps. The default k-Wave color map displays zero as white, positive pressures as yellows to reds to black, and negative pressures as light to dark blue-greys (see `getColorMap`).

```
% run the simulation
sensor_data = kspaceFirstOrder2D(kgrid, medium, source, sensor);
```

As the function runs, status updates and computational parameters are printed to the command line.

```
Running k-Wave simulation...
  start time: 22-Feb-2017 13:33:04
  reference sound speed: 1500m/s
  dt: 20ns, t_end: 12.06us, time steps: 604
  input grid size: 128 by 128 grid points (12.8 by 12.8mm)
  maximum supported frequency: 7.5MHz
  smoothing p0 distribution...
  calculating Delaunay triangulation...
  precomputation completed in 0.67051s
  starting time loop...
  estimated simulation time 3.2012s...
  simulation completed in 3.5675s
  total computation time 4.305s
```

When the time loop has completed, the function returns the recorded time series at each of the sensor points defined by `sensor.mask`. This is indexed as `sensor_data(sensor_point_index, time_index)`. For a Cartesian sensor mask, the time series are returned in the same order as the Cartesian coordinates specified in `sensor.mask`. A visualisation of the sensor data recorded in this example is given below.

```
% plot the simulated sensor data
figure;
imagesc(sensor_data, [-1, 1]);
colormap(getColorMap);
ylabel('Sensor Position');
xlabel('Time Step');
colorbar;
```
